# Novel compound heterozygous *PIEZO1* variants in dehydrated hereditary stomatocytosis initially suspected as myelodysplastic syndromes: a case report

**DOI:** 10.3389/fonc.2025.1574518

**Published:** 2025-05-21

**Authors:** Yongcheng Sun, Tao Wang, Shuyan Wang, Yan Chen, Zhijuan Xu, Cong Shi, Zanzan Wang, Guifang Ouyang

**Affiliations:** ^1^ Department of Hematology, The First Affiliated Hospital of Ningbo University, Ningbo, Zhejiang, China; ^2^ Zhejiang Key Laboratory of Digital Technology in Medical Diagnostics, Dian Diagnostics Group Co., Ltd., Hangzhou, Zhejiang, China; ^3^ Department of Histopathology, Ningbo Clinical Pathology Diagnosis Center, Ningbo, Zhejiang, China

**Keywords:** PIEZO1, compound heterozygous, allo-HSCT, DHS, case report

## Abstract

**Introduction:**

Dehydrated hereditary stomatocytosis (DHS) is a rare autosomal dominant congenital non-immune hemolytic anemia caused by pathogenic variants in the *PIEZO1* gene. Its clinical presentation often overlaps with other hematological disorders, leading to diagnostic challenges and potential mismanagement.

**Case presentation:**

A 22-year-old man presented with a 7-year history of anemia initially misdiagnosed as myelodysplastic syndrome (MDS) due to hypercellular bone marrow findings and MDS-like features. Over time, his condition progressed to include cerebral venous sinus thrombosis (CVST), a severe complication. Comprehensive genetic testing at our hospital using whole-exome sequencing (WES) revealed novel compound heterozygous *PIEZO1* variants: NM_001142864.4: c.6622A>G (p.Ile2208Val) and NM_001142864.4: c.3160C>A (p.Leu1054Met). These findings confirmed the diagnosis of DHS. The patient underwent allogeneic hematopoietic stem cell transplantation (allo-HSCT), resulting in resolution of his hematological abnormalities and symptoms.

**Conclusion:**

This case underscores the importance of considering DHS in patients with unexplained anemia and MDS-like features, particularly when associated with thrombotic complications. It highlights the critical role of genetic testing in diagnosing rare hereditary anemias and demonstrates that allo-HSCT can be a curative treatment in selected cases.

## Introduction

Dehydrated hereditary stomatocytosis (DHS; OMIM: #194380) is an autosomal dominant congenital non-immune hemolytic anemia characterized by the presence of stomatocytes—abnormal red blood cells (RBCs) that exhibit a unique mouth-like shape (1). The prevalence of DHS is not well-defined due to its rarity, with estimates suggesting it may occur in approximately 1 in 8,000 to 1 in 50,000 individuals, depending on the population studied (1, 2). DHS primarily arises from variants in the *PIEZO1* gene, which encodes a mechanosensitive ion channel that is essential for maintaining cellular ion balance in RBCs (3). Pathogenic variants in *PIEZO1* frequently produce a gain-of-function channelopathy, leading to delayed channel inactivation and heightened permeability to monovalent cations, particularly sodium and calcium (3–5). This dysregulation eventually disrupts normal osmoregulatory mechanisms of RBCs and contributes to the formation of stomatocytes.

DHS can often be misdiagnosed as other hemolytic disorders due to overlapping clinical features and laboratory findings (6–10). Both DHS and various forms of hemolytic anemia present with nonspecific symptoms such as anemia, jaundice, and splenomegaly, making it challenging to differentiate among them based solely on clinical presentation. Furthermore, the characteristic stomatocytes seen in DHS may be mistaken for other abnormal red blood cell morphologies found in conditions like hereditary spherocytosis or myelodysplastic syndromes (MDS) (8–10). The subtle differences in red blood cell indices and the presence of additional cytopenias can further complicate the diagnostic process. As a result, clinicians may overlook the specific genetic basis of DHS, relying instead on more common diagnoses. This misdiagnosis can lead to inappropriate treatments and delays in obtaining the correct diagnosis, which may exacerbate symptoms and increase healthcare costs (10). Notably, splenectomy, often considered for symptom management for immune-mediated hemolytic anemia, has been shown to be ineffective in DHS and is associated with a significantly increased risk of thromboembolic complications (11). Therefore, it is essential to conduct comprehensive evaluations and genetic testing in suspected cases to ensure accurate diagnosis of DHS.

Here, we report a 22-year-old man who has been misdiagnosed as MDS for 7 years mainly due to hypercellular bone marrow (BM) findings and overlapping clinical symptoms that obscured the accurate diagnosis of DHS. This misdiagnosis led to a gradual worsening of his condition, resulting in symptoms that evolved from initial anemia to cerebral venous sinus thrombosis (CVST), a potentially life-threatening complication. By whole-exome sequencing (WES), we identified novel compound heterozygous *PIEZO1* variants consisting of the maternally inherited NM_001142864.4: c.6622A>G (p.Ile2208Val) and the paternally inherited NM_001142864.4: c.3160C>A (p.Leu1054Met) in this patient. The patient was ultimately diagnosed with DHS and cured by BM transplantation.

## Case presentation

### MDS-like features in the past medical history

In March 2023, a 22-year-old man with 7 years history of anemia visited our hospital for a second medical opinion regarding his health condition. At age 15, he was found to have anemia (hemoglobin 89 g/L, mean corpuscular volume [MCV] 108 fL) during a routine health examination. Despite further investigations, no obvious underlying cause for these abnormalities was found. The patient was initially prescribed folic acid and vitamin B12 supplementation, but his symptoms showed minimal improvement.

Over the following three months, the patient’s condition gradually worsened, with increasing fatigue and recurrent jaundice. He then underwent a comprehensive hematological examination at a local hospital, where he was tentatively diagnosed with MDS. The peripheral smear showed that a small proportion (9%) of RBCs appeared elliptical. The serum levels of ferritin, folic acid and vitamin B12 were within normal range. Additional tests, including the Coombs test, hemoglobin electrophoresis, methemoglobin reduction test (MRT), and osmotic fragility test (OFT), all yielded normal results. BM aspirate showed hypercellularity with an elevated myeloid/erythroid (M/E) ratio of 5.54:1. The myeloid lineage showed active proliferation, particularly with an increased proportion of neutrophil precursors. The overall cell morphology in the BM appeared normal, without evidence of atypical cellular features. Multiparameter flow cytometry of BM aspirate detected less than 1% blasts, with an immunophenotype positive for CD34, CD38, CD58, myeloperoxidase (MPO), and HLA-DR. Chromosomal analysis of BM cells showed a normal male karyotype (46, XY), and fluorescence *in situ* hybridization (FISH) for common chromosomal abnormalities associated with MDS returned negative results. Based on these findings, MDS was suspected at the local hospital, but no further treatment was initiated.

### The development of CVST

Ten months prior to visiting us, the patient began to experience unexplained persistent pain in the left temporal region of brain, accompanied by periods of dizziness and sweating. Subsequently, the patient sought further medical attention at the same hospital as before. A complete blood count (CBC) revealed significant anemia (red blood cell count 1.62 × 10^^12^/L, hemoglobin 60 g/L) and a slightly increased platelet count of 366 × 10^^9^/L. Coagulation parameters were not available. Imaging studies, including magnetic resonance imaging (MRI) and cerebral angiography, showed evidence of CVST, with multiple thrombotic lesions observed in the bilateral transverse sinuses and bilateral sigmoid sinuses. Upon the diagnosis of CVST, the patient received anticoagulation therapy, catheter-directed endovascular thrombolysis, balloon venoplasty and symptomatic treatment. After 10 days of intensive treatment, his headaches and dizziness subsided, and his neurological function remained intact. Upon discharge, the patient was prescribed long-term oral medications, including Warfarin, ferrous succinate, folic acid, and vitamin C.

### Final diagnosis of DHS

To pinpoint the exact cause of his ongoing symptoms and complex clinical presentation, the patient sought further assistance at our hospital. On admission, physical examination revealed pallor (conjunctival and palmar), mild scleral icterus, and tachycardia (heart rate 112 bpm). No hepatosplenomegaly was confirmed by abdominal palpation and ultrasound. Neurological examination showed no focal deficits or signs of meningeal irritation. A comprehensive review of the patient’s medical history raised suspicion for an underlying hematological abnormality that could explain his condition, prompting us to proceed with a detailed re-evaluation.

**Figure 1:**
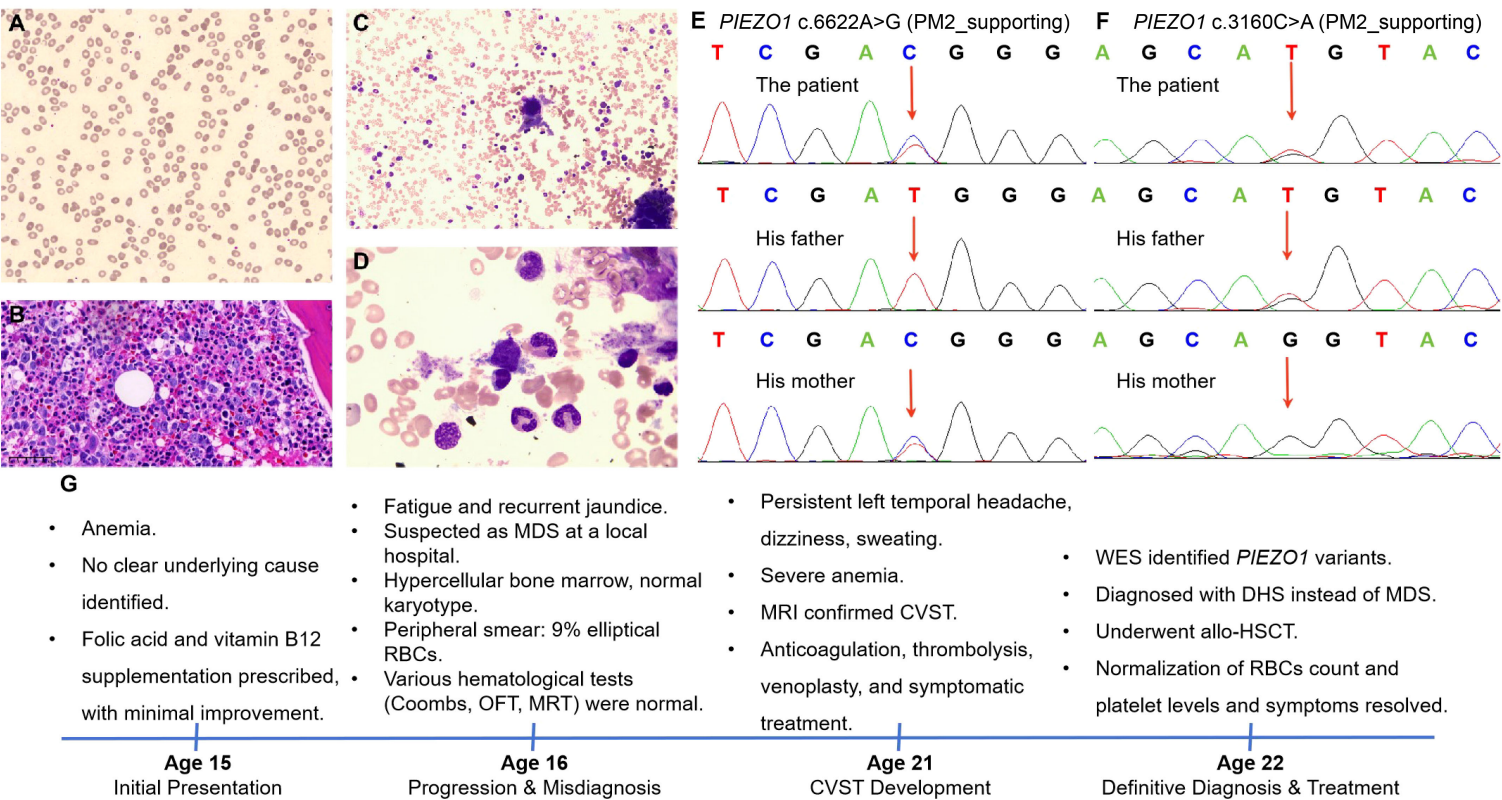
Integrated diagnostic findings in the patient of dehydrated hereditary stomatocytosis (DHS). **(A)** Peripheral blood smear showing erythrocyte morphology in the patient with dehydrated hereditary stomatocytosis (Wright-Giemsa, 20X). **(B)** Bone marrow biopsy shows that the marrow is markedly hypercellular. The granulocyte-to-erythroid ratio is slightly increased. Granulocytic cells at all stages are present, with a mild increase in immature cells, primarily at the promyelocyte and earlier stages. Eosinophils are easily observed. Erythroid cells are relatively rare, mainly at the polychromatic and orthochromatic erythroblast stages. Megakaryocytes are increased and scattered, with both small, hypolobulated forms and large, hyperlobulated forms observed. **(C, D)** Morphology of bone marrow cells derived from the patient (C, 20X; D, 50X). **(E, F)** The Sanger sequencing results, illustrating *PIEZO1* NM_001142864.4: c.6622A>G **(E)** and the paternally inherited NM_001142864.4: c.3160C>A **(F)** in the investigated family. **(G)** Timeline of main clinical events. MDS, myelodysplastic syndrome; RBCs, red blood cells; OFT, osmotic fragility test; MRT, methemoglobin reduction test; MRI, magnetic resonance imaging; CVST, cerebral venous sinus thrombosis; allo-HSCT, allogeneic hematopoietic stem cell transplantation.

In consistent with previous results, our blood laboratory testing revealed severe anemia (**Table 1**). Coagulation tests revealed increased PT, APTT, TT, and INR, while other coagulation indexes remained within the normal range (**Table 1**). It is important to note that the patient was receiving anticoagulation therapy at the time of evaluation, which may have influenced the coagulation parameters. Notably, the peripheral smear again found abnormal appearance of RBCs (shown in **Figure 1A**). BM examinations revealed previous MDS-like features, characterized by hypercellularity (M/E ratio: 4.52:1) and marked megakaryocyte hyperplasia (shown in **Figures 1B, C**). These megakaryocytes exhibited various abnormalities, showing signs of dysplasia and abnormal maturation (shown in **Figure 1D**). Immunohistochemistry (IHC) performed on the BM biopsy showed positive staining for CD34, CD117, MPO, and lysozyme. A targeted NGS panel for myeloid neoplasms (covering 248 genes, **Supplementary Table S1**) was performed on BM-derived DNA using the Illumina NovaSeq platform. The mean sequencing depth was 1000×, with >95% of target regions covered at ≥500×. No pathogenic or likely pathogenic variants were detected in this panel. Given the patient’s complex clinical picture, WES was further performed to investigate potential genetic causes. The WES analysis identified novel compound heterozygous *PIEZO1* variants, specifically NM_001142864.4: c.6622A>G (p.Ile2208Val) and NM_001142864.4: c.3160C>A (p.Leu1054Met). Parental testing by the Sanger sequencing confirmed that these variants were inherited in a compound heterozygous fashion, with the NM_001142864.4: c.6622A>G variant inherited from the mother and the NM_001142864.4: c.3160C>A variant inherited from the father (shown in **Figures 1E, F**). The patient’s parents were both healthy with no history of hematological diseases. Following genetic confirmation, the patient was diagnosed with DHS, which explained the longstanding anemia, thrombocytosis, and MDS-like features observed in this patient.

**Table 1:** Key hematologic parameters at diagnosis of DHS.

Parameter (unit)	Results	Normal range
Red blood cells count (10^12/L)	2.2	4.3~5.8
RDW (%)	14.6	12.2~14.6
Reticulocyte count (10^12/L)	4.01	0.5~1.5
Hemoglobin (g/L)	81	130~175
Hematocrit (%)	24	40~50
MCV (fL)	112	82~100
MCH (pg)	37	27~34
MCHC (g/L)	332	316~354
Platelet count (10^9/L)	329	125~350
Total bilirubin (μmol/L)	9.5	≤26
Ferritin (ng/mL)	170.9	23.9~336.2
Vitamin B12 (pmol/L)	227	133.02~675.45
Folic acid (nmol/L)	54.37	>9.08
Lactic dehydrogenase (U/L)	135	125~250
PT (s)	24.7	9.4~12.5
INR	2.28	0.90~1.20
FIB (g/L)	2.2	2.0~4.0
APTT (s)	45.5	25.1~36.5
TT (s)	16.9	10.3~16.6
D-dimer (ng/mL)	45	0.0~243.0

RDW, red cell distribution width; MCV, mean corpuscular volume; MCH, mean corpuscular hemoglobin; MCHC, mean corpuscular hemoglobin concentration; PT, prothrombin time; INR, international normalized ratio; FIB, fibrinogen; APTT, activated partial thromboplastin time; TT, thrombin time.

### Outcome and follow-up

The patient successfully underwent allogeneic hematopoietic stem cell transplantation (allo-HSCT), which resulted in significant improvement in his hematological parameters. Post-transplantation, the patient showed normalization of his red blood cell count and platelet levels, and his symptoms of fatigue and jaundice resolved (**Figure 1G**). The CVST, which had complicated his clinical course, did not recur following transplantation. The patient is currently under regular follow-up to monitor for any potential complications or relapse.

## Discussion

This case underscores the complexity of diagnosing rare hematological conditions when clinical features overlap with multiple potential diagnoses. The patient, a 22-year-old man with a longstanding history of anemia and MDS-like features, was ultimately diagnosed with DHS resulting from compound heterozygous *PIEZO1* variants. These variants, a novel missense variant (p.Ile2208Val) in exon 45 and another missense mutation (p.Leu1054Met) in exon 22, were identified as the underlying cause of the patient’s persistent hematological abnormalities. To the best of our knowledge, both variants are described for the first time in the *PIEZO1* gene. The c.6622A>G (p.Ile2208Val) and c.3160C>A (p.Leu1054Met) variants were also not found in the population database (gnomAD v4.0). Experimental evidence assessing their impact on protein function may be lacking, and computational predictions offer conflicting assessments of their effect on protein structure or function. Consequently, both variants were classified as variants of uncertain significance (VUS; PM2_supporting) under ACMG guidelines. Importantly, the patient’s parents were asymptomatic carriers, which may suggest that one of the variants in this compound heterozygous pair is not pathogenic. While the possibility of reduced penetrance or variable expressivity cannot be excluded, the compound heterozygous state is more likely to account for the observed manifestations. Further functional studies are needed to clarify the pathogenicity and genotype-phenotype correlations of these variants.

The patient’s clinical presentation of persistent anemia and MDS-like BM appearance, including hypercellularity and increased myeloid/erythroid ratio, is suggestive of MDS. However, the absence of blasts, the lack of chromosomal abnormalities, and negative FISH results ultimately excluded MDS as the definitive diagnosis. The diagnosis of DHS can be further challenging, particularly when the characteristic stomatocytes are rare or difficult to detect on peripheral blood smears. In our case, only a small proportion of abnormal red blood cells with stomatocytic features were observed, making the initial diagnosis elusive. Osmotic gradient ektacytometry is a valuable and often critical diagnostic tool for confirming DHS (12). This technique measures the deformability of red blood cells under varying osmotic conditions, and in DHS, it typically reveals a leftward shift of the osmolarity curve, which is indicative of erythrocyte dehydration. However, osmotic gradient ektacytometry was not available at our hospital, which posed a challenge in the diagnostic process. The discovery of *PIEZO1* mutations through WES was crucial in resolving the diagnostic ambiguity and establishing the diagnosis of DHS, which explains both the hematological abnormalities and the thrombophilic tendencies observed in the patient. Notably, genetic testing not only resolved the diagnostic uncertainty but also highlighted the importance of considering inherited red blood cell disorders in cases with unexplained anemia and ambiguous BM findings. Given the evolving understanding of germline predisposition in hematological disorders, recent study underscores the necessity of incorporating germline genetic assessments into the diagnostic workup of young patients with suspected MDS (13). Expanding access to comprehensive genetic testing and promoting awareness of its diagnostic value are vital in preventing misdiagnoses and ensuring appropriate management for these patients.

The occurrence of CVST in our patient reflects the well-documented prothrombotic risk associated with hemolytic disease. DHS is characterized by erythrocyte dehydration and chronic hemolysis, both of which contribute to a hypercoagulable state. The ongoing hemolysis releases adenosine diphosphate (ADP) from platelets and other cellular sources, which in turn promotes platelet aggregation and thrombus formation (14). Additionally, hemolysis leads to the depletion of nitric oxide (NO), a critical mediator that inhibits platelet activation and promotes vasodilation (14). The reduction in NO bioavailability further exacerbates endothelial dysfunction, creating a favorable environment for thrombosis (14). The management of CVST in DHS presents significant challenges due to the complex interplay between thrombosis risk and the underlying hemolytic disorder. In our case, timely initiation of anticoagulation and catheter-directed thrombolysis led to favorable outcomes without recurrence of thrombotic events. However, it is crucial to maintain close monitoring for potential bleeding complications, especially in patients with severe anemia and thrombocytopenia.

Allo-HSCT is rarely performed for DHS but offers curative potential in patients with severe and refractory disease. The decision to proceed with HSCT in this patient was based on a comprehensive risk-benefit analysis. Key factors influencing this decision included: (1) the patient’s young age (22 years), which may optimize his candidacy for tolerating HSCT-associated risks such as graft-versus-host disease (GVHD) and infections; (2) the lack of effective alternatives, as splenectomy—contraindicated in DHS due to thrombotic risks—was not viable, and supportive care alone could not address the underlying genetic defect; and (3) the presence of transfusion-dependent anemia, which significantly impaired the patient’s daily functioning and quality of life. In our patient, transplantation led to a complete resolution of hematological abnormalities and cessation of thrombotic complications, underscoring the efficacy of this approach. While HSCT carries inherent risks, the benefits of cure in this context outweighed potential adverse outcomes, emphasizing the importance of individualized assessment that balances disease severity, genetic etiology, and patient-specific factors.

In conclusion, this case highlights the diagnostic challenges posed by overlapping clinical features in rare hematological disorders and underscores the importance of comprehensive genetic evaluation in cases with persistent hematological abnormalities of uncertain etiology. DHS, although rare, should be considered in the differential diagnosis of chronic hemolytic anemia, particularly when patients exhibit a history of thrombosis, stomatocytic red blood cells, or refractory anemia with MDS-like features. This report also expands the genotypic spectrum of DHS, describing two novel *PIEZO1* variants that further emphasize the genetic diversity underlying this condition.

## Data Availability

The original contributions presented in the study are included in the article/**Supplementary Material**, Further inquiries can be directed to the corresponding author.
